# Reliability and accuracy of smartphones for paediatric infectious disease consultations for children with rash in the paediatric emergency department

**DOI:** 10.1186/s12887-019-1416-8

**Published:** 2019-01-31

**Authors:** İlker Devrim, Mine Düzgöl, Ahu Kara, İlknur Çağlar, Fatma Devrim, Nuri Bayram, Hurşit Apa

**Affiliations:** 1 Department of Pediatric Infectious Diseases, Dr. Behçet Uz Children’s Hospital, İzmir, Turkey; 2 Department of Pediatrics, Dr. Behçet Uz Children’s Hospital 22, İzmir, Turkey; 3 Department of Pediatric Emergency Department, Dr. Behçet Uz Children’s Hospital, İzmir, Turkey

## Abstract

**Objective:**

Smartphones and associated messaging applications have become the most common means of communication among health care workers and the general population. The aim of this study was to evaluate the reliability and accuracy of smartphones for the diagnosis of rash in children admitted to emergency departments during the night shift.

**Methods:**

The images of the children who were admitted to the paediatric emergency department with rash were included in this study, and at least two images taken with smartphones by residents or paediatric infectious disease fellows were re-directed to the chief consultant of the Paediatric-Infectious Department via smartphone. Initial diagnosis by the consultant was recorded, and the patient’s physical examination was performed by another clinician on the first working day; diagnostic tests were planned by this clinician. The definitive diagnosis was recorded and compared with the initial diagnosis.

**Results:**

Among the 194 patients, the most common final diagnoses were chickenpox (varicella-zoster infections) in 33 patients (17.0%) and skin infections (including impetigo, ecthyma, erysipelas and cellulitis) in 33 patients (17.0%). The initial diagnosis, which was performed via WhatsApp on a smartphone, was identical to the final diagnosis in 96.3% of the cases. Incompatible initial diagnoses included 4 measles cases, 1 staphylococcal scalded skin syndrome case, 1 cutaneous leishmaniasis case and 1 petechial rash case.

**Conclusions:**

Our study has shown that the use of a smartphone-based instant messaging application for transmitting images of paediatric rash is accurate and useful for diagnosis. However, physical examination and medical history are still the primary methods. Consultation via smartphones in emergency departments for paediatric rashes during nightshifts would help both clinicians and patients.

## Background

The use of smartphones and associated messaging applications, including one of the most popular applications, WhatsApp (WhatsApp Inc., Mountain View, Calif, acquired by Facebook Inc.), in medicine has been increasing and has become the most common way of communicating among health care workers [1]. Smartphones are indispensable in today’s world, and current studies, including marketing surveys, have reported that up to 84% of physicians in the United States use smartphones for professional purposes, and 81% of United Kingdom health care professionals use smartphones [2, 3]. As technology develops, improved smartphones with high-resolution cameras and high-speed internet connections with wide coverage make sharing photos and videos via applications easier and faster. One of the successful and most commonly used applications is WhatsApp (WhatsApp Inc., Mountain View, Calif, acquired by Facebook Inc), which has reached 900 million users, 64 billion messages, and 600 million pictures per day [1].

Smartphones have been widely used for educational purposes in residency and fellowship programmes. Residents carry smartphones to help increase mobility and multitasking abilities [4]. Additionally, smartphones shorten consultation times and reduce time lost in the decision tree procedure compared to that of beepers, resulting in an improvement in quality of patient care. In the residency programme of paediatrics and paediatric infectious disease fellowship programmes, smart phones might be useful for the differential diagnosis and treatment of children with fever and rash in which early and accurate diagnosis is important.

The aim of this study was to evaluate the reliability and accuracy of diagnoses performed remotely by a consultant for children who were admitted to the paediatric emergency department (ED) during the night shift with the presentation of rash. Images of the lesions were captured and sent via smartphones using the instant messaging application WhatsApp (WhatsApp Inc., Mountain View, Calif, acquired by Facebook Inc.) and the image diagnosis was compared with that of the final diagnosis.

## Methods

**Fig. 1:**
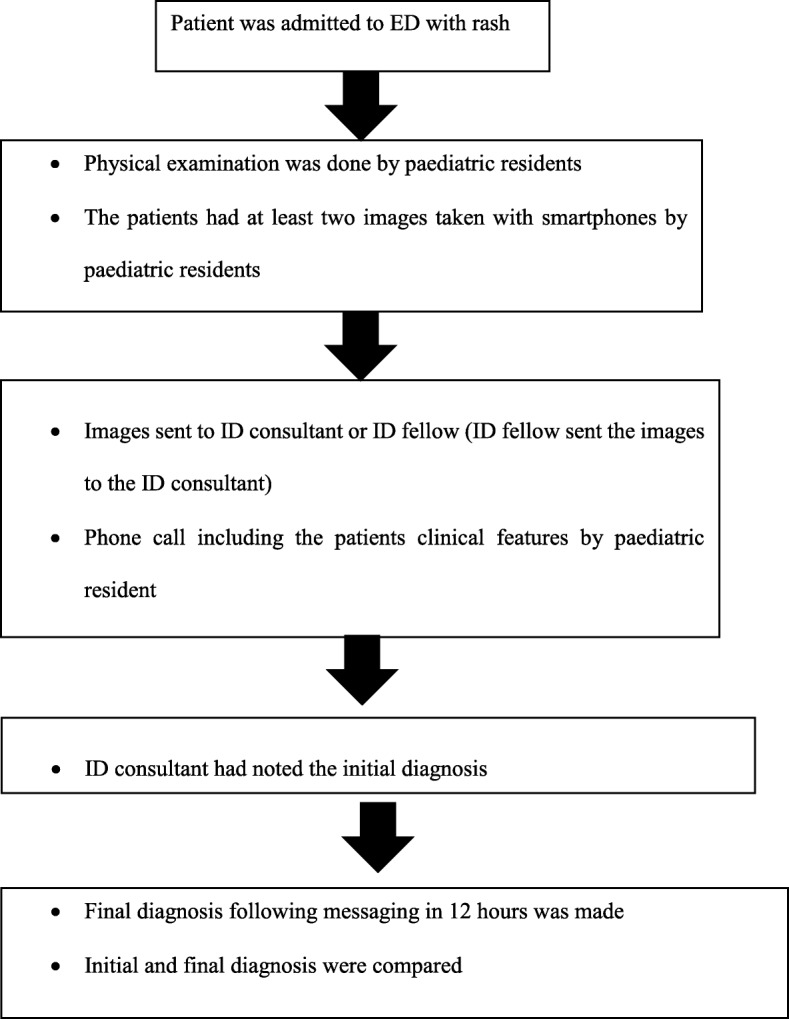
The algorithm of the study

The study was carried out in the Pediatric Infectious Disease and Pediatric Emergency Department of Dr. Behçet Uz Children’s Hospital from January 2015 to January 2017. Dr. Behçet Uz Children’s Hospital, Izmir, is a 400-bed paediatric teaching hospital with 128,000 paediatric emergency visits, 15,000 hospitalizations and 750,000 outpatient visits annually. The images of the children who were admitted to the ED with rash were included in the study. The patients had at least two images taken with smartphones by paediatric residents, which were re-directed to the Pediatric Infectious Disease Department consultant’s smartphone (iPhone 6, Apple Inc., Cupertino, Calif) via WhatsApp instant messaging application by either the paediatric residents or indirectly through paediatric disease fellows. Additionally, a phone call with verbal information, including demographic and epidemiologic features, medical history, and laboratory results (including limited information such as complete blood count and basic biochemical tests) obtained during routine consultations performed during the night shift, was conducted with the consultant. The initial diagnosis by the consultant was recorded, and the patient’s physical examination was performed by another clinician unaware of the diagnosis by the consulting clinician within the following 12 h of messaging. The images of the patients whose physical examination was not performed within 12 h were not included in the study. The second diagnosis of the patients after physical examination was recorded, and the definitive diagnosis after the diagnostic tests, including serological and molecular diagnostic tests, was recorded (Fig. 1).

Data were analysed with SPSS, version 19.0 (IBM company, US). The descriptive analysis was performed using frequencies and quartiles. Cohen’s kappa was used to determine if there was agreement between the two clinicians’ diagnoses. The study was approved by the local ethical committee of the institution. Written informed consent was obtained from participants and the parents, and assurance was given that original images would not be used anywhere else by the investigators.

## Results

A total of 249 patients were enrolled in the study: 19 patients who had only one suboptimal image and 16 patients who did not come for treatment or were lost to follow up were excluded. Twenty patients who had come for treatment 12 h past onset were also excluded. The remaining 194 images were included in the study. Among the 194 patients, 58 (29.9%) were females and 136 (70.1%) were males. The mean age was 58,1 ± 45,0 months (ranging from 23 days to 16 years).

Among the 194 patients, the most common final diagnoses were chickenpox (varicella-zoster infections) in 33 patients (17.0%) and skin infections (including impetigo, ecthyma, erysipelas and cellulitis) in 33 patients (17.0%), followed by shingles (herpes zoster infections) in 13 patients (6.7%), insect bite in 9 patients (4.6%) and herpes-simplex infections in 7 patients (3.6%). There are 10 patients (5.1%) with “non-specific” exanthems who had no definite diagnosis after the diagnostic tests were done and reviewed in the miscellaneous diagnosis part.

The final diagnoses of the patients are reviewed in Table 1. The initial diagnosis, which was performed via WhatsApp on the smartphone, showed 96.3% compatibility with the final diagnosis. Cohen’s kappa was used to determine if there was agreement between the two clinicians’ diagnoses of 194 children with rashes. There was almost perfect agreement between two clinicians’ diagnoses of the children, K = 0,944, *p* < 0.005.

**Table 1:** The distribution of the final diagnosis of the patients

Causes	Number of patients (*N*)
Herpesviridae infections	
Chickenpox	33
Zona zoster	13
Roseola infantum	5
Herpes simplex virus infections	13
Other viral infections	
Epstein-Barr-Virus	3
Erythema Infectiosum	6
Rubella	1
Measles	1
Hand-foot-and-mouth disease	9
Superficial skin infections and cellulitis	
Impetigo and pyodermitis	17
Scarlet fever	6
Cellulitis and erysipelas	15
Paronychia	3
Kerion	4
Parasitosis and fungal infections	
Scabies	5
Cutaneous leishmaniasis	3
Necrotizing cellulitis and subcutaneous tissue infection	
Erythema multiforme major	10
Steven-Johnson Syndrome/ Toxic epidermal necrolysis	4
Purpura fulminans	2
Non-infectious diseases	
Allergic reactions	18
Insect bite	10
Erythema nodosum	1
Miscellaneous^a^	12
TOTAL	**194**

^a^İncludes 1 sun burn,1 Lupus vulgaris and 10 “non-specific” exanthems whose etiology was not classifed despite laboratory tests

Incompatible preliminary diagnoses included 4 measles cases, 1 staphylococcal scalded skin syndrome case, 1 cutaneous leishmaniasis case and 1 petechial rash case. The suspected measles cases were in patients 5 to 36 months of age, and further serological and molecular diagnostic methods, including polymerase chain reaction, excluded the diagnosis. In three of the four patients, no aetiology of the rash was determined. The initial diagnosis of one patient whose face was severely erythematous and oedematous with crusted and purulent lesions associated with low-grade fever was staphylococcal scalded skin syndrome; the child was hospitalized in the Paediatric Infectious Disease Ward, however, a re-evaluation of the child’s medical history and physical examination revealed that the child had a sunburn injury due to excessive sun exposure on the beach while the family left him sleeping. The final diagnosis of one patient with diffuse petechia localized on her right leg was diagnosed as a jellyfish sting from her medical history. The last patient was a 4-year-old Syrian patient who had an ulcerated lesion on her forehead; the initial diagnosis was cutaneous leishmaniasis. However, the histopathological and bacteriological investigations revealed a diagnosis of lupus vulgaris.

## Discussion

In the medical field of paediatrics, smartphones are widely used. The spectrum of use of smartphones is wide and includes assessing neonatal jaundice, using checklists for paediatric emergency guidelines, managing of diarrhoeal diseases in resource-limited settings and utilizing smartphone-integrated electrocardiograms, in addition to educational purposes for paediatric trainees [4–8]. In this study, we evaluated the clinical diagnostic accuracy of images of paediatric patients sent via the WhatsApp smartphone application by consultants from the paediatric ED during the night shift.

The compatibility rate of diagnoses via WhatsApp in this study was 96.3%. To our knowledge, this is the first study focusing on the utility of smartphones for the consultation and diagnosis of paediatric rash in the paediatric ED. Hubiche et al. evaluated adult and paediatric patients who had taken photos of their skin lesions with their smartphone during outpatient visits [9]. In this study, a total of 162 patients were included, of which paediatric patients formed the majority of the patients, and photography of the lesions via smartphones was found to be useful, especially in patients without skin lesions at the time of the clinical visit [9]. In this study, the photographs of the lesions were taken by the patients using their own smartphones; however, in our study, the photographs were taken by the residents who might be more selective in choosing the best characteristic lesions (if there was more than one lesion) of the patients, which might increase the accuracy.

Since the last decade, overcrowding of paediatric EDs has become an important issue, and rash comprises a considerable amount of ED visits. The rapid differential diagnosis of the rash is very important for the early treatment of patients, such as in cases of meningococcemia, protecting other patients in the emergency room and taking precautions for preventing transmission, such as in the case of measles. In this study, the patients with rash or skin lesions had been diagnosed with a variety of diseases, including infectious and non-infectious diseases, and consultation via smartphone successfully helped the rapid diagnosis during night shifts. However, consultation via smartphones resulted in the misdiagnosis of 4 patients with an incorrect diagnosis of measles. A clinical case definition for measles has been developed for epidemiological purposes; however, due to its nature, measles can be difficult to distinguish from other febrile exanthems, such as rubella, roseola, erythema infectiosum, human herpesvirus 6, Epstein-Barr Virus infection and drug eruptions. Thus, confirmation of the diagnosis by laboratory testing has become routine, and laboratory results had excluded the diagnosis of measles. A possible diagnosis of measles resulted in isolation precautions in these patients, which might be the most reasonable strategy for protecting other patients and health care workers in EDs before the exclusion of measles.

In our study, 5% of the patients etiological diagnoses could not be found after evaluation of morphology and laboratory investigations, and were defined as “non-specific exanthema”. In one study from Italy including 112 adult and child patients, 36 (32%) of the patients had no specific diagnosis after investigations, supporting an older study including only children reporting 35% of the children remained undiagnosed [10, 11]. The difference in the rates of undiagnosed “non-specific” exanthems can change according to the geographical features, seasonal changes, vaccination programs, vaccination coverage and the availability of the diagnostic tests. In our study, most of the incompatible preliminary diagnoses were diagnosed as “non-specific” exanthems after investigations. In the clinical settings in which “non-specific” exanthems are more common, it would not be wrong to suggest that the agreement level between clinician’s diagnoses would not be as high as in our study.

One of the important issues with smartphone-based instant messaging application for transmitting images is the protection of personal data. According to the firms own website, WhatsApp’s end-to-end encryption provides unique lock and key for recipients and message sender [12], however the debate about the concerns of security of patients confidentiality are increasing. Finally, National Health service of England had advised officially that WhatsApp should never be used for the sending of information in the professional healthcare environment [13]. Moreover there are also legal regulations within General Data Protection Regulation in the European Union [14]. In our clinical practice, this kind of application is fast and useful, and still used in our country extensively, enabling fast communications between doctors. However new security standards for WhatsApp or new applications for health professionals for sharing data should be developed [15]. In our study, no personal data such as name, surname and identifying features were shared via WhatsApp, and personal data was shared via phone call between health professionals.

There are some limitations to this study. The data were retrospectively collected from the medical consultation forms and no additional information about the quality of the images, the technical features of the cameras of the smartphone or the smart phone brand was available. Characteristics of skin lesions or rashes might change after photos are taken via smartphone as time passes; however, a maximum of 12 h was set as the maximum time interval between the initial diagnosis to the physical examination by the paediatric infectious disease specialist. To our knowledge, this is the first study on the utility of WhatsApp via smartphones for the differential diagnosis of rash in paediatric EDs.

## Conclusions

Our study has shown that the use of a smartphone-based instant messaging application for transmitting images of paediatric rash is accurate and useful for differential diagnoses. However, physical examination and medical history taking are still the primary methods of evaluation of paediatric rash. Due to its advantages, such as its speed and reduction of wait times, instant messaging services and smartphones might be one of the complementary components of paediatric infectious disease consultations in EDs, especially during night shifts.

## Abbreviation

ED: Emergency department
